# Knowledge towards Prevention and Early Detection of Chronic Kidney Disease and Associated Factors among Hypertensive Patients at a Chronic Illness Clinic of Jimma Town Public Hospitals

**DOI:** 10.1155/2020/5969326

**Published:** 2020-10-21

**Authors:** Belachew Tegegne, Tigist Demeke, Shemsedin Amme, Afework Edmealem, Sewunet Ademe

**Affiliations:** ^1^Department of Nursing, School of Nursing and Midwifery, Wollo University, Dessie, Ethiopia; ^2^Department of Nursing, Jimma University, Ethiopia

## Abstract

**Background:**

Morbidity and mortality due to chronic kidney disease are increasing among hypertensive patients in Sub-Saharan Africa. The majority of hypertensive patients with chronic kidney disease are not diagnosed at an early stage because of poor knowledge. However, to the best of our knowledge, there is no study conducted in Ethiopia about knowledge of hypertensive patients towards prevention and early detection of chronic kidney disease. Thus, the aim of this study was to assess knowledge towards prevention and early detection of chronic kidney disease and associated factors among hypertensive patients at Jimma town public hospitals, Ethiopia.

**Methods:**

A hospital-based cross-sectional study was conducted among 332 hypertensive patients using an interviewer-administered questionnaire and medical record reviewing from April 5 to May 21, 2019. Study participants were selected using simple random sampling. Data were collected by using a standardized questionnaire. Data were entered into Epidata version 3.1 and analyzed by SPSS version 23. Descriptive statistics and bivariable and multivariable logistic regression were applied. To identify factors, a 95% confidence level and *P* value of less than 0.05 were considered.

**Results:**

Over half (59.6%) were males, and the mean (±SD) age of participants was 54.92 (12.91) years. Among the total participants, more than half of them (47.9%) had good knowledge. Attending secondary education (AOR = 2.9, *P* = 0.014), higher education (AOR = 5.4, *P* = 0.001), working in private sectors (AOR = 4.3, *P* = 0.001), taking three and above drugs per day (AOR = 0.55, *P* = 0.016), and having a family history of kidney disease (AOR = 2.3, *P* = 0.012) were significantly associated with knowledge. *Conclusion and Recommendation*. Near to half of the study participants had good knowledge towards prevention and early detection of chronic kidney disease. Attending secondary education and above, working in private sectors, taking three and above drugs per day, and having a family history of kidney disease were independent predictors of knowledge. Hypertensive patients should be encouraged to be aware of risk factors of CKD, and health care providers should educate hypertensive patients about the prevention and early detection of chronic kidney disease.

## 1. Introduction

Uncontrolled hypertension (HTN) is the primary risk factor for chronic kidney disease, coronary artery disease, stroke, heart failure, and peripheral artery disease [1, 2]. Chronic kidney disease (CKD) is defined as the presence of kidney damage or glomerular filtration rate (GFR < 60 mL/min per 1.73m^2^) greater than 3 months [3, 4]. The normal GFR is 125 mL/min/1.73 m^2^, and there are five stages of CKD: Stage 1 if GFR ≥ 90 mL/min/1.73 m^2^ (kidney damage with normal or increased GFR), Stage 2 if GFR = 60–89 mL/min/1.73 m^2^ (mild decrease in GFR), Stage 3 if GFR = 30–59 mL/min/1.73 m^2^ (moderate decrease in GFR), Stage 4 if GFR = 15–29 mL/min/1.73 m^2^ (severe decrease in GFR), and Stage 5 if GFR < 15 mL/min/1.73 m^2^ (end-stage renal disease) [4, 5]. Patients with end-stage renal disease (ESRD) need lifelong dialysis or kidney transplantation to survive for the rest of their life [5, 6].

Nowadays, developing countries including Ethiopia are disproportionally affected by both communicable and noncommunicable diseases (double burden disease) [7]. In Ethiopia, some institution-based studies revealed that the prevalence of HTN ranges from 16.9 to 27.4% [8, 9]. Hypertension is the second leading risk factor for CKD; it occurs in 67% to 92% of patients with CKD, with increased prevalence as renal function declines [10]. Hypertension accounts for 28% of CKD causes, and the treatment of HTN among CKD patients is challenging because of the bidirectional cause and effect relationship [11].

In Sub-Saharan Africa (SSA), the prevalence of CKD in the general population is 13.2% [12] and the prevalence of CKD among hypertensive and diabetic patients is 34.5% and 24.7%, respectively [3]. In Ethiopia, the prevalence of CKD is increasing alarmingly among diabetic and hypertensive patients. Some institution-based studies indicated that the prevalence of CKD among hypertensive and diabetic patients ranges from 18.2% to 26% [13–15].

In most of SSA, patients cannot get treatment for kidney failure because of huge cost, shortage of skilled health care providers, and limited service settings for renal replacement therapy. As a result, patients' quality of life is decreased and health expenditure is increased; ultimately, premature death can occur [16]. Likewise, in Ethiopia, patients cannot afford and access for renal replacement therapies because of huge cost, limited dialysis centers, and nonexistence of kidney transplantation centers.

The majority of patients with renal dysfunction are not aware of having CKD until 90% of kidney function becomes lost [17]. Because of this, most early stages of CKD are underdiagnosed; patients come to health facilities when the disease is in an advanced stage which gives opportunity for the progression of disease [18]. Preventive strategies of CKD include identifying high-risk individuals, awareness creation for patients to prevent renal disease, public education, enhancing awareness of policymakers and health care providers, blood and urine tests to detect early stages of CKD, and modifying the lifestyle of susceptible individuals like hypertensive patients [12, 19].

Limited studies were conducted regarding knowledge towards prevention and early detection of CKD in SSA [20–23]. However, to the best of our knowledge, there is no study conducted in Ethiopia about knowledge of hypertensive patients towards prevention and early detection of CKD. Thus, the aim of this study was to assess knowledge towards prevention and early detection of CKD and associated factors among hypertensive patients at the chronic illness clinic of Jimma town public hospitals, Southwest Ethiopia.

## 2. Methods

### 2.1. Study Area and Period

The study was conducted in Jimma town public hospitals from April 5 to May 21, 2019. Jimma town is located 352 kilometers far apart from Addis Ababa, the capital city of Ethiopia. Jimma University Medical Center and Shenen Gibie Hospital are public hospitals in Jimma town. Jimma University Medical Center is a teaching hospital with 659 beds and serves more than 18 million people. Shenen Gibie Hospital is a general hospital. Chronic illness care is one of the services in which both hospitals provide to the community within and outside Jimma town. According to 2019 registration, there were a total of 1520 hypertensive patients in Jimma University Medical Center and Shenen Gibie Hospital.

### 2.2. Study Design

A hospital-based cross-sectional study was conducted.

### 2.3. Population

All hypertensive patients who had to follow-up at Jimma University Medical Center and Shenen Gibie Hospital chronic illness follow-up clinic were the source population. Hypertensive patients who had to follow-up at Jimma University Medical Center and Shenen Gibie Hospital chronic illness follow-up clinic during the data collection period were taken as the study population.

### 2.4. Eligibility Criteria

All adult (age ≥ 18 years) hypertensive patients and registered in the list of hypertensive patients until the commencement of data collection were included in the study. On the contrary, hypertensive patients who were already diagnosed with CKD obtained from medical record reviewing and seriously ill patients were excluded.

### 2.5. Sample Size and Sampling Technique

The sample size was determined using a single population proportion formula by considering 1.96 for the standard normal variable with a 5% level of significance (*α* value), 95% confidence interval, 5% margin of error, and 50% proportion. Because of the absence of a previous study, 50% is taken as a proportion. Since the source population is less than 10,000, a correction formula was utilized. By adding a 10% nonresponse rate, the final sample size was 338. Simple random sampling (lottery method) was used to recruit study participants using their medical record number. After that, participants were interviewed and their medical records are reviewed when they came for medication refill and medical checkup at a chronic illness follow-up clinic.

### 2.6. Dependent Variable

The dependent variable is the knowledge towards prevention and early detection of CKD.

### 2.7. Independent Variables

The independent variables are sociodemographic characteristics (age, sex, residence, educational status, marital status, occupation, and monthly income) and clinical factors (duration of hypertension, number of comorbidities (other than HTN), number of drugs, family history of kidney disease, family history of diabetes mellitus, and family history of cardiac disease).

### 2.8. Operational Definition

Knowledge: awareness of hypertensive patients about CKD and measured by calculating the mean score of 24 items.

Good knowledge: if participants scored ≥mean score of knowledge questions.

Poor knowledge: if participants scored <mean score of knowledge questions.

Prevention: range of health promotion actions that are geared towards limiting the development of CKD.

Number of drugs: drugs taken by hypertensive patients for a long period of time and classified as <3 and ≥3 drugs per day.

### 2.9. Data Collection Tools and Procedure

The data were collected by a structured interviewer-administered questionnaire and medical record reviewing using data abstraction format. The questionnaire had three parts. The first part was about the sociodemographic characteristics of participants. The second part assessed clinical factors, and the third part was the chronic kidney disease screening index tool. The knowledge questionnaire was adopted from the CKD screening index which was validated, and its reliability was checked in the previous studies. In the previous studies, its internal consistency was 0.87 and 0.8 in Palestine [18] and Jordan [24], respectively. In this study, the reliability of the tool was a Cronbach's alpha of 0.78 which is above the acceptable value. Knowledge scale was measured using a dichotomous scale composed of 24 items. Knowledge score ranged from 0 to 24 (1 = yes, 0 = no, and unsure) [25]. The questionnaire was translated into local languages (Afan Oromo and Amharic) then back to English by another person to maintain consistency. After that, data were collected by four professional nurses and two supervisors and data collectors made rapport with patients before commencing the interview.

### 2.10. Data Quality Assurance

The questionnaire was pretested on 5% of the sample at Agaro Hospital to check acceptability and consistency two weeks prior to actual data collection. After reviewing the result of the pretest, modification of the questionnaire was performed for clarity, sensitiveness, and completeness of the questionnaire. Training was given for data collectors and supervisors for one day on the objective of the study. Data collectors were fluent speakers of local languages (Afan Oromo and Amharic). Continuous supervision and daily checking of the collected data were performed by investigators.

### 2.11. Data Processing and Analysis

The collected data was numerically coded, checked, and entered into Epidata version 3.1 and then exported to SPSS version 23.0 for analysis. All independent variables having *P* value < 0.25 in the bivariable analysis were fitted into a multivariable logistic regression to control for the possible effects of confounding. Then, multivariable logistic regression using the backward likelihood ratio method was done to see the associations between outcome and independent variables. Odds ratio and 95% CI were used to identify the presence and strength of association, and *P* value < 0.05 was declared as statistically significant at the final model. Model fitness was checked by the Hosmer-Lemeshow test.

## 3. Result

### 3.1. Sociodemographic Characteristics of Participants

Out of a total of 338 hypertensive patients, 332 participated in the study yielding a response rate of 98.2%. More than half, 198 (59.6%), of participants were males. The mean (±SD) age of participants was 54.92 (±12.91) years, 204 (61.4%) were living in urban, and 233 (70.2%) were married (Table 1).

### 3.2. Clinical Factors

More than one-third, 125 (37.7%), of hypertensive patients had lived with the disease between five and ten years, and half, 167 (50.3%), of hypertensive patients were treated by one or two drugs. Three hundred three (91.3%) patients had no comorbidities, and 279 (84%), 289 (87%), and 303 (91.3%) had no family history of kidney disease, diabetes mellitus, and cardiac disease, respectively (Table 2).

### 3.3. Knowledge of Hypertension Patients

After computing the answers of 332 participants, normal distribution was checked using a histogram and the distribution was normal. The mean (±SD) score of knowledge among study participants was 11.18 (±4.64). Participants who scored ≥11.18 were classified as having good knowledge, and participants who scored <11.18 were classified as having poor knowledge. Based on this, 173 (52.1%) of participants had poor knowledge and 159 (47.9%) had good knowledge. The majority (69.3%) of the study participants did not know the function of the kidney. Only near to one-fourth of the respondents (23.5%) knew that the kidney releases hormones to regulate blood pressure. With regard to risk factors of CKD, 222 (66.9%) of the respondents reported that being old is a risk factor. Being a smoker is reported as risk factors for CKD by half (50.6%) of the respondents. Moreover, having kidney stones and recurrent urinary tract infections, being obese, and having high blood pressure were reported as risk factors by 180 (54.2%), 197 (59.3%), and 195 (58.7%) of participants, respectively. However, most (78.6%) of the participants did not know the relationship between CKD and having a family history of CKD. One hundred forty (42.2%) of participants knew the importance of checking creatinine and serum urea nitrogen tests. Near to three-fourth, 257 (77.4%), of participants identified swollen feet and ankle and puffiness around the eye as symptom/sign of CKD. Most, 221 (66.6%), did not know that the final stage of CKD (ESRD) needs dialysis as a lifelong treatment (Table 3).

### 3.4. Factors Associated with Knowledge

In bivariable logistic regression, eleven variables including age, residence, marital status, educational status, occupation, monthly income, hypertension duration, taking a number of drugs, having comorbidities, family history of kidney, and cardiac disease were significant at *P* value < 0.25 and entered into multivariable logistic regression. However, in multivariable logistic regression analysis, only educational status, occupation, taking three and above drugs per day, and having a family history of kidney disease showed significant association with good knowledge at *P* value < 0.05. Participants who had attended higher education were 5 times more likely to have good knowledge as compared to participants with informal education (AOR = 5.4, 95% CI (2.23, 13.02)) (Table 4).

## 4. Discussion

It is crucial for hypertensive patients to discover the level of knowledge to develop health education to prevent major complications including chronic kidney disease. Thus, the current study was aimed at assessing the knowledge of hypertension patients towards prevention and early detection of chronic kidney disease.

### 4.1. Knowledge about Chronic Kidney Disease

In this study, 47.9% (95% CI: 42.6, 53.2%) had good knowledge about chronic kidney disease. This finding was higher than studies done in Tanzania 38.5% [22], Nigeria 27.1% [23], and Malaysia 26.6% [26]. This discrepancy across countries might be due to the difference in study design and tool. For example, a study conducted in Tanzania and Nigeria used a community-based cross-sectional study design. In addition, a study conducted in Nigeria and Malaysia used an open-ended questionnaire and a self-administered questionnaire, respectively.

However, this finding was lower than a study done in Jordan [24], where more than half of participants correctly answered 80% of knowledge questions. This might be due to the educational level of participants; in Jordan, 46% of participants had attended high school and above whereas in this study, 38.3% of participants had no formal education, less devoted time with patients by health care providers, lack of organized hypertensive education programs, and less participation of media in awareness creation in our setting.

In this study, being old is reported as a risk factor by 66.9% of the participants. This was in line with a study conducted in Jordan (66.6%) [24]. More than half (58.7%) of the participants identified hypertension as a risk factor for CKD. This was similar to a study done in Palestine (61.2%) [18]. However, it was higher than a study done in Nigeria, 43.6% [23]. This difference might be due to a difference in study design and tool. A study conducted in Nigeria was a community-based study design.

Educational status was significantly associated with knowledge towards prevention and early detection of chronic kidney disease. Accordingly, participants who had attended secondary education and above were three times and five times, respectively, more likely to have good knowledge as compared to participants who had no formal education. This finding was supported by other studies conducted in Palestine, Nigeria, Australia, Malaysia, and Singapore [18, 23, 26–28]. This could be explained by participants who had attended secondary education and above that have a chance of exposure to different educational materials like internet, manuals, magazines, leaflets, and social media. Moreover, a high level of education facilitates good communication between health care providers and patients, and they can grasp the information communicated with health care providers.

Occupational status was associated with knowledge. The current finding showed that those participants who had worked in private/NGOs were four times more likely to have good knowledge as compared to farmers. This finding was consistent with a study done in Cameron [20], where rural residents had less awareness on their renal status, and Singapore [28], where nonprofessionals had poor knowledge. In this study, also, one-fourth (25.6%) were farmers, and this might be because most farmers are illiterate and living in a rural area in the Ethiopian context. Because of iliteracy, farmers could not access health information.

In this finding, a number of drugs had a significant association with knowledge. Participants who had taken three or more drugs a day were 45% less likely to have good knowledge as compared to participants who had taken less than three drugs. The possible explanation could be pill burden, and side effects might compromise patients' adherence to their medication and might stop taking medication.

Another, having a family history of kidney disease was associated with good knowledge. Participants who had a family history of kidney disease were two times more likely to have good knowledge as compared to those participants who had no family history of kidney disease. This finding was in line with a study done in Australia [27]. This could be explained as participants who had a family history of kidney disease might have exposure to health care providers when they went to health facilities with their families and got information from them. Additionally, those patients who had family history of CKD might have information about the severity of the disease so they dig out different sources to prevent the occurrence of CKD.

### 4.2. Limitation of the Study

Since the study design is cross-sectional, it did not show a temporal relationship between cause and effect. Knowledge was assessed by an interview technique; this might lead to recall bias. Another is that comparison of study findings was held with other countries because of the limitation of literature on this topic in Ethiopia, where the health facility setup, health policy, and socioeconomic status of the population might differ across the country.

## 5. Conclusion and Recommendation

In conclusion, the current study showed that near to half of the study participants had good knowledge towards prevention and early detection of chronic kidney disease. Attending secondary education and above, working in private sectors, taking three and above drugs per day, and having a family history of kidney disease were independent predictors of knowledge. Hypertensive patients should be encouraged to be aware of risk factors of CKD, and health care providers should educate hypertensive patients about the prevention and early detection of chronic kidney disease.

## Figures and Tables

**Table 1:** Frequency distribution of sociodemographic characteristics of hypertensive patients at a chronic follow-up clinic in JTPH, June 2019 (*N* = 332).

Variable	Category	Frequency	Percent
Sex	Male	198	59.6
	Female	134	40.4

Age category	18-40 years	47	14.2
	41-60 years	181	54.5
	≥61 years	104	31.3

Residence	Urban	204	61.4
	Rural	128	38.6

Marital status	Single	35	10.5
	Married	233	70.2
	Widowed	33	9.9
	Divorced	31	9.4

Educational status	No formal education	127	38.3
	Primary [1–8]	111	33.4
	Secondary [9–12]	38	11.4
	Higher education and above	56	16.9

Occupation	Government employee	75	22.6
	Private/NGO	39	11.8
	Merchant	29	8.7
	Daily laborer	13	3.9
	Housewife	69	20.8
	Farmer	85	25.6
	Retirement	22	6.6

Monthly income (birr)	<1000	92	27.7
	1000-5000	192	57.8
	>5000	48	14.5

**Table 2:** Frequency distribution of clinical factors among hypertensive patients at a chronic follow-up clinic in JTPH, June 2019 (*N* = 332).

Variables	Category	Frequency	Percent
Hypertension duration	<1 year	62	18.7
	1-4 years	107	32.2
	5-10 years	125	37.7
	>10 years	38	11.4

Number of drugs	<3	167	50.3
	≥3	165	49.7

Number of comorbidities	None	303	91.3
	≥1	29	8.7

Family history of kidney disease	Yes	53	16.0
	No	279	84.0

Family history of diabetes mellitus	Yes	43	13.0
	No	289	87.0

Family history of cardiac disease	Yes	29	8.7
	No	303	91.3

**Table 3:** Frequency distribution of participant responses to knowledge questions in the CKD screening index at a chronic follow-up clinic of JTPH, June 2019 (*N* = 332).

Items	Yes, *n* (%)	No, *n* (%)	Unsure, *n* (%)
Kidneys regulate body water and chemicals in the blood such as sodium, potassium, phosphorus, and calcium	102 (30.7)	127 (38.3)	103 (31.0)
Kidneys remove drugs and toxins introduced into the body	128 (38.6)	124 (37.3)	80 (24.1)
Kidneys release hormones into the blood to regulate BP, produce red blood cells, and promote strong bones	78 (23.5)	127 (38.3)	127 (38.3)
CKD is a serious illness	253 (76.2)	59 (17.8)	20 (6.0)
CKD is an irreversible illness	203 (61.1)	102 (30.7)	27 (8.1)
Becoming old will decrease the function of the kidneys	222 (66.9)	87 (26.2)	23 (6.9)
Having increased BP makes me more likely to get CKD	195 (58.7)	89 (26.8)	48 (14.5)
Having DM makes me more likely to get CKD	148 (44.6)	102 (30.7)	82 (24.7)
Having a family member with CKD will increase the chances of getting CKD	71 (21.4)	158 (47.6)	103 (31.0)
Having high lipid in the blood will increase the chances of getting CKD	158 (47.6)	81 (24.4)	93 (28.0)
Being a smoker increases the chances of getting CKD	168 (50.6)	107 (32.2)	57 (17.2)
Becoming an obese person will increase the chances of getting CKD	197 (59.3)	73 (22.0)	62 (18.7)
Having untreated anemia will increase the chances of getting CKD	113 (34.0)	126 (38.0)	93 (28.0)
Certain procedures like cardiac catheterization & CT scan that require injection of drugs increase the chances of getting CKD	78 (23.5)	107 (32.2)	147 (44.3)
Having kidney stones and recurrent urinary tract infection increases the chances of getting CKD	180 (54.2)	75 (22.6)	77 (23.2)
Routine checkup of lab tests like creatinine and serum urea nitrogen will decrease chances of getting CKD	140 (42.2)	92 (27.7)	100 (30.1)
Having CKD results trouble in concentration	145 (43.7)	117 (35.2)	70 (21.1)
Having CKD consequences sleeping trouble	205 (61.7)	81 (24.4)	46 (13.9)
Having CKD brings muscle cramps at night	150 (45.2)	111 (33.4)	71 (21.4)
Having CKD results in swollen feet and ankles & puffiness around the eyes in the morning	257 (77.4)	46 (13.9)	29 (8.7)
Having CKD makes skin dry and itchy	138 (41.6)	144 (43.4)	50 (15.1)
CKD increases the amount of urination	204 (61.4)	90 (27.2)	38 (11.4)
There are five stages of CKD, and every stage needs a management plan	67 (20.2)	121 (36.4)	144 (43.4)
People in the final stage of CKD need dialysis as a lifelong treatment	111 (33.4)	90 (27.1)	131 (39.5)

**Table 4:** Multivariable logistic regression analysis of factors associated with knowledge of hypertensive patients towards prevention and early detection of CKD in JTPH, June 2019.

Variable	Category	Knowledge		COR (95% CI)	AOR (95% CI)	*P* value
		Good	Poor			
Age	18-40 years	29	18	1.70 (0.88, 3.28)^∗^		
	41-60 years	88	93	1		
	≥61 years	42	62	0.72 (0.44, 1.17)^∗^		
Residence	Urban	111	93	1.99 (1.27, 3.13)^∗^		
	Rural	48	80	1		
Marital status	Single	21	14	1.71 (0.83, 3.52)^∗^		
	Married	109	124	1		
	Widowed	15	18	0.95 (0.46, 1.97)		
	Divorced	14	17	0.94 (0.44, 1.99)		
Educational status	Informal Edu	46	81	1	1	
	Grade 1-8	48	63	1.34 (0.80, 2.26)	1.23 (0.69, 2.19)	0.492
	Grade 9-12	24	14	3.02 (1.42, 6.40)^∗^	2.9 (1.23, 6.62)	0.014
	Higher education	41	15	4.81 (2.41, 9.63)^∗^	5.4 (2.23, 13.02)	0.001
Occupation	Gov't employee	43	32	2.74 (1.44, 5.21)^∗^	1.17 (0.51, 2.67)	0.711
	Private/NGO	27	12	4.58 (2.03, 10.4)^∗^	4.3 (1.81, 10.07)	0.001
	Merchant	18	11	3.33 (1.39, 7.99)^∗^	2.57 (0.99, 6.68)	0.052
	Daily laborer	6	7	1.75 (0.54, 5.68)	1.50 (0.43, 5.23)	0.521
	Housewife	26	43	1.23 (0.63, 2.39)	1.01 (0.5, 2.05)	0.980
	Farmer	28	57	1	1	
	Retirement	11	11	2.04 (0.79, 5.27)^∗^	1.51 (0.53, 4.26)	0.44
Monthly income	<1000	38	54	0.77 (0.46, 1.26)		
	1000-5000	92	100	1		
	>5000	29	19	1.66 (0.87, 3.16)^∗^		
HTN duration	<1 year	31	31	0.94 (0.51, 1.73)		
	1-4 year	47	60	0.73 (0.44, 1.24)^∗^		
	5-10 year	65	60	1		
	≥10 years	17	21	0.76 (0.37, 1.58)		
Number of drugs	<3	90	77	1	1	
	≥3	69	96	0.62 (0.40, 0.95)^∗^	0.55 (0.34, 0.89)	0.016
No. comorbidity	None	142	161	1		
	≥1	17	12	1.61 (0.74, 3.48)^∗^		
Fx. kidney disease	Yes	33	20	2.004 (1.1, 3.7)^∗^	2.3 (1.20, 4.47)	0.012
	No	126	153	1	1	
Fx. cardiac disease	Yes	18	11	1.88 (0.86, 4.12)^∗^		
	No	141	162	1		

Fx: family history; ^∗^*P* value < 0.25; 1: reference category; COR: crude odds ratio; AOR = adjusted odds ratio; Hosmer-Lemeshow test = 0.10.

## Data Availability

The datasets used and/or analyzed during the current study are available from the corresponding author on reasonable request.

## Abbreviations

AOR: Adjusted odds ratio
CKD: Chronic kidney disease
ESRD: End-stage renal disease
HTN: Hypertension
JTPH: Jimma town public hospitals
SD: Standard deviation
SSA: Sub-Saharan Africa.
